# Engineered mesenchymal stem cell–derived extracellular vesicles overexpressing miR-146a alleviate neuroinflammation in Alzheimer’s disease

**DOI:** 10.4103/NRR.NRR-D-25-00404

**Published:** 2025-09-29

**Authors:** Jia Zhang, Siqi He, Qiguo Xiao, Weijie Jiang, Bo Wang, Jing Lu, Hongfeng Gu, Yajin Liao, Zhi Wang, Ying Xu, Dan Wang, Xiaoqing Tang, Ling Qi

**Affiliations:** 1 The Second Affiliated Hospital, University of South China, Hengyang, Hunan Province, China; 2 Division of Gastroenterology, Institute of Digestive Disease, the Affiliated Qingyuan Hospital of Guangzhou Medical University, Qingyuan People’s Hospital, Qingyuan, Guangdong Province, China; 3 Department of Physiology & Key Laboratory of Hunan Province for Major Brain Diseases, Hengyang Medical School, University of South China, Hengyang, Hunan Province, China; 4 The First Affiliated Hospital, Institute of Anesthesiology, University of South China, Hengyang, Hunan Province, China; 5 Guangdong-Hong Kong-Macau Institute of Central Nervous System Regeneration, Key Laboratory of Central Nervous System Regeneration, Ministry of Education, Jinan University, Guangzhou, Guangdong Province, China; 6 Department of Pathology, Beihua University, Jilin, Jilin Province, China

**Keywords:** Alzheimer’s disease, amyloid-β, cognitive function, mesenchymal stem cell–derived extracellular vesicles, microglia, miR-146a, neuroinflammation, neuroprotection, nuclear factor-κB, nuclear receptor subfamily 4 group A member 3 (NR4A3)

## Abstract

**Figure:**
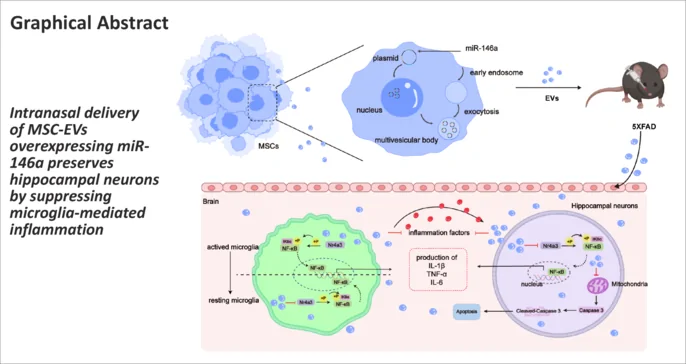

Alzheimer’s disease is an inflammatory neurodegenerative disease for which no effective clinical treatment currently exists. We have previously reported that mesenchymal stem cell–derived extracellular vesicles delay retinal degeneration by exerting anti-inflammatory effects though the miR-146a–nuclear receptor subfamily 4 group A member 3 axis; however, it remains unclear how NR4A3 drives inflammation. Herein, we engineered mesenchymal stem cell–derived extracellular vesicles overexpressing miR-146a to explore their possible neuroprotective effects and the underlying mechanisms in both cell and animal models of Alzheimer’s disease. In HT22 cells co-cultured with lipopolysaccharide-induced RAW264.7/BV2 cells, extracellular vesicles overexpressing miR-146a significantly reduced the number of apoptotic cells and inhibited proinflammatory cytokine expression, nuclear factor (NF)-κB activation, and caspase-3/apoptosis regulator BAX signaling. These effects of extracellular vesicles overexpressing miR-146a were replicated in 5×FAD mice. In addition, extracellular vesicles overexpressing miR-146a inhibited the activation of microglia and astrocytes, reduced amyloid-β and phosphorylated tau expression, lowered the number of apoptotic cells in the hippocampus, and improved the cognitive function of these Alzheimer’s disease model mice. Mechanistically, miR-146a negatively regulated the expression of nuclear receptor subfamily 4 group A member 3 and suppressed the expression of proinflammatory cytokines and nuclear factor-κB signaling. Furthermore, NR4A3 overexpression promoted nuclear factor-κB and proinflammatory cytokine expression as well as nuclear factor-κB signaling. The upregulation of NR4A3 and the inflammatory response was reversed by miR-146a overexpression. Finally, NR4A3 was identified as a transcriptional activator of nuclear factor-κB using chromatin immunoprecipitation polymerase chain reaction. Collectively, these findings indicate that extracellular vesicles overexpressing miR-146a may alleviate the progression of Alzheimer’s disease by exerting anti-inflammatory effects via the NR4A3–nuclear factor-κB axis. They are thus a potential therapeutic candidate for the clinical treatment of neurodegenerative diseases.

## Introduction

Alzheimer’s disease (AD) is a neurodegenerative disease that is characterized by cognitive impairment. Several widely accepted hypotheses for the pathogenesis of AD exist, including the amyloid-β (Aβ) cascade hypothesis, the cholinergic hypothesis, the abnormal phosphorylation of tau hypothesis, the neuroinflammation hypothesis, and the metal ion disorder hypothesis (Heneka et al., 2015; Lv et al., 2020; Ju et al., 2022). To date, fundamental and clinical studies have intensively focused on the detrimental effects of Aβ accumulation and neurofibrillary tangles on neuronal function (Ulamek-Koziol et al., 2013). Thus, clinical drug discovery for AD has mainly been limited to cholinesterase inhibitors and N-methyl-D-aspartate receptor-blockers. Although some Aβ inhibitors have been investigated recently, none have successfully stopped or reversed cognitive decline in phase III clinical trials (Ulamek-Koziol et al., 2013; Van Der Kant et al., 2020). For example, from 2022 to 2023, the pharmaceutical giants Roche and Eli Lilly successively announced the failure of gantenerumab and solanezumab (two clinical drug candidates for AD) in phase III clinical trials. Therefore, new and effective clinical drugs for AD need to be urgently explored.

AD is characterized by the secondary death of neurons, leading to the loss of neuronal function. Neuroinflammation is reportedly strongly correlated with secondary neuronal death (Verbakel et al., 2018). Neuroinflammation manifests as reactive morphological changes and functional abnormalities in glial cells such as microglia and astrocytes (Lampron et al., 2013). Active microglia can directly damage or kill neurons (Hickman et al., 2018). Microglia have been observed to proliferate in the hippocampal region of AD mice (Streit et al., 1999; Soulet and Rivest, 2008), accompanied by clear morphological changes (e.g., the retraction of microglial processes and enlargement of cell bodies). Simultaneously, inflammatory factor expression is abnormally increased (Lampron et al., 2013; Liu et al., 2020). The Aβ-induced inflammatory cascade further activates astrocytes and microglia (Akiyama et al., 2000; Iaccarino et al., 2016). Combating neuroinflammation in the AD brain may therefore be a good entry point for the clinical treatment of neurodegenerative diseases.

Stem cell–derived exosomes are extracellular vesicles (EVs) that are 30–150 nm in diameter and are secreted by stem cells. They mainly constitute lipids, proteins, and nucleic acids (Sato-Kuwabara et al., 2015). Compared with mesenchymal stem cell (MSC) transplantation, MSC-derived EVs (MSC-EVs) show low immunogenicity, can be easily stored and administered, and mitigate the risk associated with stem cell transplantation (Weng et al., 2022). Preclinical research has indicated that MSC-EVs may be useful as a treatment for a variety of illnesses, and can easily cross the blood–brain barrier (Gong et al., 2020; Xiao et al., 2021; Zhai et al., 2022). We have previously reported that MSC-EVs effectively promote the survival of retinal neurons in retinitis pigmentosa mice and inhibit neuroinflammation without causing appreciable adverse effects (Zhang et al., 2022). Because neuroinflammation is an important pathological characteristic of AD, we hypothesized that MSC-EVs may also exert neuroprotective effects in the brains of AD mice.

Many drugs have been reported to have anti-inflammatory effects; however, few are effective in central nervous system diseases because they are obstructed by the blood–brain barrier. The advantages of EVs (nanoscale, low immunogenicity, ease of storage and administration) led us to decide to engineer MSC-EVs. We have previously identified multiple functional microRNAs (miRNAs) in MSC-EVs through miRNA sequencing, and demonstrated that miR-146a-5p is an effective component of stem cell therapy (Zhang et al., 2022). In addition, miR-146a expression is reported to be significantly altered in the blood or cerebrospinal fluid of AD patients (Calvayrac et al., 2015; Maffioletti et al., 2019; Lei et al., 2021; Gong and Sun, 2022). We therefore engineered MSC-EVs to overexpress miR-146a in the present study. miR-146a-5p can target the proinflammatory transcription factor nuclear receptor subfamily 4 group A member 3 (*NR4a3*) in microglia, thereby reducing the expression of proinflammatory cytokines and suppressing the nuclear factor (NF)-κB signaling pathway (Zhang et al., 2022). However, the mechanisms by which NR4A3 regulates NF-κB expression, proinflammatory factor expression, and the NF-κB signaling pathway remain obscure.

*NR4a3* is a member of the *NR4a* nuclear receptor family, and has been reported to enhance interleukin (IL)-1β-induced NF-κB activation in osteoarthritis (Ma et al., 2020). Conversely, NR4A3 inhibits inflammation in vascular smooth muscle cells by suppressing NF-κB activation (Calvayrac et al., 2015). Notably, whether NR4A3 exerts anti-inflammatory or proinflammatory effects in the brains of AD patients remains unclear. In the present study, we prepared normal MSC-EVs (NC-EVs) and MSC-EVs overexpressing miR-146a (OE-EVs). We then established an *in vitro* co-culture neuroinflammation model comprising hippocampal neurons cultured with macrophages or microglia under lipopolysaccharide (LPS) stimulation, and used a mouse model of AD (five familial AD mice, or 5×FAD mice) to explore the possible neuroprotective effects of OE-EVs and the underlying mechanisms. The OE-EVs inhibited neuroinflammation by regulating NF-κB signaling via the transcription factor NR4A3, promoted neuronal survival, and improved cognitive function in the AD model. These results suggest that OE-EVs may be an effective clinical treatment for brain dysfunction and neuronal damage in AD.

## Methods

### Animals

Male 5×FAD mice were purchased from Guangdong Youdu Co., Ltd. (Guangzhou, China; license No. SCXK (E) 2021-0025; specific-pathogen-free grade), and C57BL/6J control mice were purchased from Guangdong Medical Lab Animal Center (Guangdong, China; license No. SCXK (Yue) 2022-0002; specific-pathogen-free grade). The 5×FAD (amyloid precursor protein [APP] K670N/M671L [Swedish] + I716V [Florida] + V717I [London] and presenilin 1 [PS1] M146L+ L286V) mouse is a good model of AD, with emphasis on the overaccumulation of Aβ and marked AD symptoms (Oakley et al., 2006).

All mice were raised in a controlled animal facility (23 ± 2°C, 60% ± 5% relative humidity) with a standard 12-hour light/12-hour dark cycle. They were allowed free access to rodent chow and water at a density of four or five mice per cage. The mice were anesthetized with 2% inhaled isoflurane (Shenzhen Ruiwode Life Technology Co., Ltd., Shenzhen, China) followed by the intranasal administration of EVs at a dose of 5 μL per mouse, with treatments administered at 48-hour intervals. All animal experiments were conducted according to the National Institutes of Health Guide for the Care and Use of Laboratory Animals and were approved by the Animal Ethics Committees at Qingyuan People’s Hospital (approval No. LAEC-2020-025, approval date July 25, 2020) and the Second Affiliated Hospital of University of South China (approval No. 2023-661, approval date November 6, 2023).

### Cell culture

RAW264.7 murine macrophages, BV-2 murine microglia, and HT22 murine neuronal cells were cultured in Dulbecco’s Modified Eagle Medium (Gibco, Grand Island, NY, USA) supplemented with 10% heat-inactivated fetal bovine serum (VivaCell, Shanghai, China) and 1% penicillin-streptomycin. Human umbilical cord MSCs were kindly provided by Dr. Guifang Zhao (Zhang et al., 2022), and were maintained in Dulbecco’s Modified Eagle Medium/F-12 (Gibco) containing 10% fetal bovine serum, 1% penicillin-streptomycin, and 10 ng/mL basic fibroblast growth factor (PeproTech, Cranbury, NJ, USA). All cell lines were incubated at 37°C in a humidified atmosphere with 5% CO_2_.

### *In vitro* cell inflammation model

RAW264.7 and BV-2 cells were seeded in six-well plates (1 × 10^6^ cells/well) and allowed to adhere for 12 hours. Following 24 hours of standard culture, the vehicle control (NC) group received phosphate-buffered saline (PBS), whereas the experimental (LPS-treated) group was stimulated with 1 μg/mL of LPS (Sigma-Aldrich, St. Louis, MO, USA, L2880) for 24 hours.

### Establishment of the Alzheimer’s disease inflammatory co-culture model

RAW264.7 (a murine macrophage cell line) or BV-2 (a murine microglia cell line) cells were seeded at 5 × 10^5^ cells/well in the upper chamber of 0.4-μm pore size Transwell® inserts (LabSelect, Anhui, China). After 24 hours of stimulation with 1 μg/mL of LPS, the inserts were transferred to six-well plates containing HT22 cells (a murine hippocampal neuronal cell line) plated at 1 × 10^6^ cells/well in the lower chamber. Co-cultures were maintained for an additional 24 hours prior to subsequent experiments.

### Cell viability assays

RAW264.7 or BV-2 cells were seeded in 96-well plates (1 × 10^4^ cells/well) and incubated for 12 hours to allow attachment. After 24 hours of stimulation with 1 μg/mL of LPS, the cells were washed three times with ice-cold PBS. Fresh medium containing 10% Cell Counting Kit-8 (CCK-8) reagent (GLPBIO, Montclair, CA, USA) was added, followed by 2 hours of incubation. Absorbance at 450 nm was then measured using a microplate reader.

### Annexin V-allophycocyanin/propidium iodide apoptosis analysis

After 24 hours of RAW264.7 or BV-2 cell stimulation with 1 μg/mL of LPS, cell supernatants were collected and co-cultured with HT22 cells. Apoptosis was then assessed using an Annexin V-allophycocyanin (APC)/propidium iodide (PI) dual-staining kit (KeyGEN Biotech, Jiangsu, China). HT22 cells were stained with 2 μL of Annexin V-APC and 2 μL of PI at 37°C for 30 minutes in the dark. Fluorescent images were captured using a confocal laser scanning microscope (ZEISS LSM 880, Oberkochen, Germany).

### Lentiviral packaging and infection

The procedure was performed according to the manufacturer’s instructions. Using the lentiviral packaging method, the target gene plasmid (with green fluorescent protein) was transferred into HEK293T cells. After 24 hours, the supernatant containing the target virus was collected. The detailed protocol was as follows. First, 10 μg of pSPAX2 (HANBI, Shanghai, China), 5 μg of pMD2G (HANBI), and 10 μg of the target plasmid (GENE, Shanghai, China) were mixed at the ratio recommended for T75 flasks. The plasmid mixture was then combined with Lipofectamine 3000 at a 1:2 ratio. The transfection complex was gently dripped onto 293T cells (used here for high-efficiency lentiviral packaging because of their superior transfection properties; the target gene was later transduced into RAW264.7, BV2, and MSC cells). Next, the transfected cells were cultured at 37°C with 5% CO_2_ for 24–48 hours. The cell culture medium was then collected and centrifuged at 2000 × *g* for 10 minutes to remove cellular debris. Finally, the clarified lentiviral supernatant was harvested for the subsequent transduction of RAW264.7, BV2, and MSC cells.

### Isolation, labeling, and identification of normal mesenchymal stem cell–derived extracellular vesicles and mesenchymal stem cell-derived extracellular vesicles overexpressing miR-146a

Ultrahigh-speed differential centrifugation was used to collect NC-EVs and OE-EVs as previously described (Zhang et al., 2022). Briefly, when the confluence of MSCs and MSCs overexpressing miR-146a (OE-MSCs) reached 80%–90%, the complete medium containing fetal bovine serum (VivaCell) was replaced with serum without EVs. After culturing for 24 hours, the supernatant was centrifuged at 2000 × *g* for 30 minutes at 4°C. Subsequently, the supernatant was centrifuged again at 10,000 × *g* for 30 minutes at 4°C to further eliminate organelles, and centrifuged one final time at 120,000 × *g* for 90 minutes at 4°C in an ultracentrifuge using an SW32Ti rotor (Beckman Coulter, Brea, CA, USA). The EV pellets were then dissolved in PBS and stored at −80°C. PKH26 (Fubaike, Beijing, China) was used to label EVs after the final centrifugation, and the mixture was centrifuged again at 120,000 × *g* for 90 minutes at 4°C. To confirm the successful isolation of exosomes, the exosomal markers cluster of differentiation (CD)9, CD63, and programmed cell death 6-interacting protein (ALIX) were detected using western blot analysis. Detailed antibody information is provided in **Additional Table 1**, and is as previously described (Zhang et al., 2022).

### Experimental groups and extracellular vesicle administration to 5×FAD mice

Building upon established experimental protocols (Zhang et al., 2024), 4-month-old 5×FAD male mice were randomly assigned to three groups: 5×FAD + PBS (*n* = 8), 5×FAD + NC-EVs (*n* = 8, 10 μg/μL of NC-EVs), and 5×FAD + OE-EVs (*n* = 8, 10 μg/μL of OE-EVs). The NC-EVs or OE-EVs were administered intranasally (5 μL every 48 hours). Each animal received 10 injections.

### Morris water maze test

The spatial learning and memory abilities of mice were tested using the Morris water maze following a previous protocol (Vorhees and Williams, 2006) with some modifications. Briefly, mice were acclimatized to the test apparatus (120 cm in diameter) 1 day before the experiment. The maze was filled with opacified water that was replaced daily, and the water temperature was maintained at 19–22°C. The maze was artificially divided into four quadrants, and the platform (10 cm in diameter) was fixed 1 cm beneath the water surface and randomly placed in the center of one quadrant. Different colored and shaped objects were mounted on the quadrant walls as landmarks. During training, the mice swam freely for 60 seconds to find the platform. Mice who failed to find the platform were guided to it and allowed to stay on it for 10 seconds. Mice were trained four times daily, and the reported data represent the average of the four trials. The platform was removed 24 hours after the last training trial, and mice were then tested for memory retention in a probe trial. The swimming activity of each mouse was monitored using a video camera mounted overhead, and was automatically recorded via EthoVision XT behavioral tracking software (Noldus, Wageningen, the Netherlands).

### Chromatin immunoprecipitation-quantitative polymerase chain reaction

Chromatin immunoprecipitation (ChIP) assays were performed according to the manufacturer’s instructions (Magnetic Beads; Cell Signaling Technology, Danvers, MA, USA). In brief, BV2 cells were fixed with 1% formaldehyde (Biosharp, Beijing, China) followed by glycine termination. The anti-NR4A3 antibody was then added and the sample was incubated overnight at 4°C with rotation. The immune complexes were isolated using Protein G/A magnetic beads (Cell Signaling Technology) before being washed three times with buffer. PCR amplifications were performed with 40 cycles (95°C for 3 minutes, 95°C for 10 seconds, and 60°C for 30 seconds). cDNA was transcribed from 1 μg of RNA using an RNA Reverse Transcription Kit (Applied Genecopia, Guangzhou, China). A First-Strand cDNA Synthesis Kit (Applied Genecopia) was used to synthesize the cDNA. The relative expression ratio of NF-κB mRNA was normalized to glyceraldehyde-3-phosphate dehydrogenase (Gapdh) expression using the ΔCt method (2^–ΔΔCt^). The quantitative PCR results were analyzed using the software provided with the real-time PCR machine (Bio-Rad, Hercules, CA, USA).

### Quantitative reverse transcription-polymerase chain reaction

Cells, EVs, and hippocampal tissue were harvested using TRIzol reagent (Sigma-Aldrich). Total RNA was extracted, and 1 mg of RNA was reverse-transcribed using the cDNA kit. The miRNA quantitative reverse transcription (qRT)-PCR Detection Kit was then used to detect the expression level of miR-146a, which was normalized to the expression level of U6. The qRT-PCR primers were purchased from GeneCopoeia Co., Ltd. (Rockville, MD, USA) and are listed in **Additional Table 2**.

### Western blot analysis

Cells, EVs, and hippocampal tissue were resuspended in radioimmunoprecipitation assay lysis buffer for 30 minutes on ice. The protein concentrations of the supernatants were measured using a Bicinchoninic Acid Protein Assay Kit (Beyotime, Zhengzhou, China). Total protein (30–50 μg) was separated using sodium dodecyl sulfate–polyacrylamide gel electrophoresis and transferred to polyvinylidene difluoride membranes (Millipore, Burlington, MA, USA). The membranes were then blocked with 5% skim milk, probed with primary antibodies overnight at 4°C on a shaker, incubated with secondary antibodies (Cell Signaling Technology) for 2 hours at 25°C on a shaker, washed three times, and visualized using a chemiluminescence system (Bio-Rad). Next, the blots were semi-quantified using ImageJ software (version 1.53e, National Institutes of Health, Bethesda, MD, USA). The antibody information is listed in **Additional Table 1**.

### Terminal deoxynucleotidyl transferase dUTP nick end labeling staining

All terminal deoxynucleotidyl transferase dUTP nick end labeling (TUNEL) staining procedures were performed using the In Situ Cell Death Detection Kit, POD (Roche Applied Science, Indianapolis, IN, USA) according to the manufacturer’s instructions. Nuclei were visualized using 4′,6-diamidino-2-phenylindole staining (1:1000; Invitrogen, Carlsbad, CA, USA). All sections were photographed under a confocal laser scanning microscope (LSM900, Zeiss, Germany).

### Tissue processing

After anesthetic overdose, mouse brains were quickly removed and placed into 4% paraformaldehyde for 24 hours at 4°C. The brains were then dehydrated in 0.01 M PBS containing 40% sucrose for 48 hours at 4°C. Next, brains were embedded in optimal cutting temperature compound and moved into a −80°C freezer. The brains were then cryo-sectioned lengthwise (20 μm per slice) using a cryostat (Leica, Germany); the sections were mounted on glass slides for further experiments.

### Immunohistochemistry

Mice brains sections were washed three times with 0.01 M PBS, incubated with primary antibodies for 6 hours at room temperature, then washed three times with 0.01 M PBS and incubated with secondary antibodies for 2 hours at room temperature. After washed three times with 0.01 M PBS, sections were covered with a glass slide. The antibody information is listed in **Additional Table 1**.

### Statistical analysis

All fluorescence data and western blot experimental results were analyzed using ImageJ software (version 1.53e) via the statistical “Integrated density” analysis. The fluorescence intensity/laser intensity used for imaging was consistent across all fluorescence data plots. All values are presented as the mean ± standard error of the mean. Unpaired two-tailed Student’s *t*-test was used to compare two groups, and one-way analysis of variance (ANOVA) with Tukey’s *post hoc* test and two-way ANOVA with a Bonferroni post hoc test were also used. All statistical analyses were performed using GraphPad Prism version 5.0 for Windows (GraphPad Software, Boston, MA, USA; www.graphpad.com). Significance was set at *P* < 0.05.

## Results

### Establishment of a co-culture system of macrophages/microglia and hippocampal neurons

**Figure 1:**
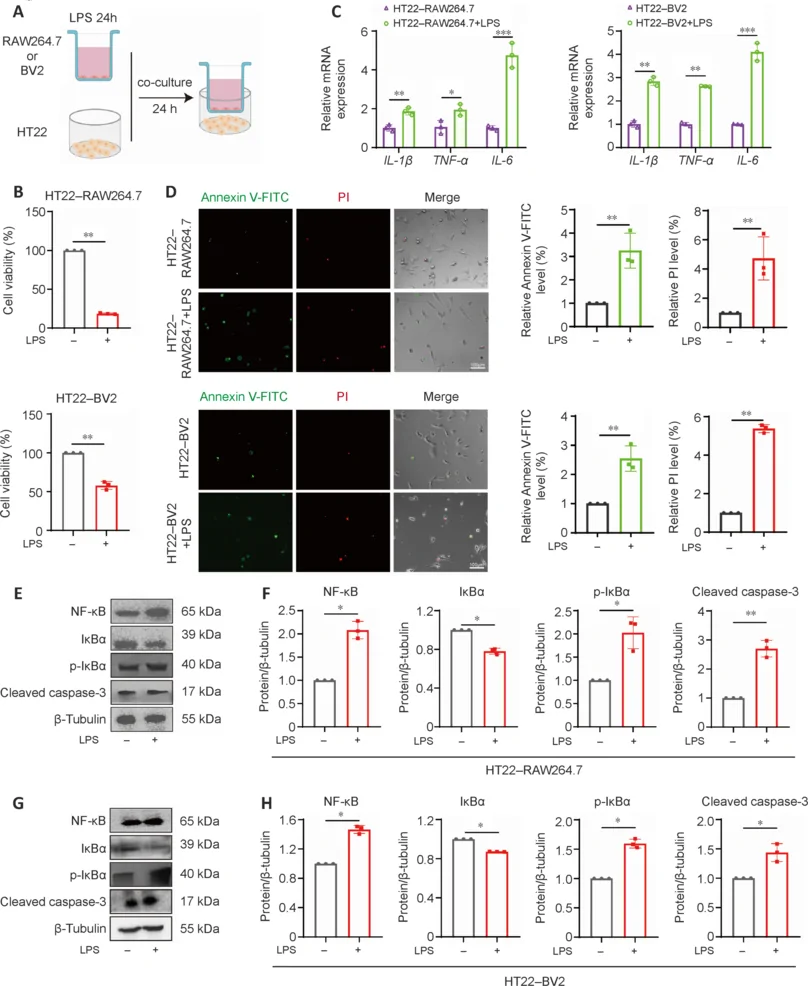
Establishment of a co-culture system of macrophages/microglia and hippocampal neurons. RAW264.7 and BV2 cells were treated with 1 μg/mL LPS for 24 hours and subsequently co-cultured with HT22 cells. (A) Schematic of the neuroinflammatory model. (B) Viability of HT22 cells in the two neuroinflammatory co-culture models. (C) mRNA expression of IL-1β, TNF-α, and IL-6 in HT22 cells, as detected by qRT-PCR. (D) Annexin V-FITC and PI staining with fluorescence quantification in the two co-culture models. Scale bars: 100 μm. (E–H) Western blot analysis of NF-κB, IκBα, p-IκBα, cleaved caspase-3, and β-tubulin in HT22 cells from the two co-culture systems. Quantitative results are shown for the HT22–RAW264.7 system (E, F) and the HT22–BV2 system (G, H). Data are presented as mean ± SEM (*n* = 3). *P < 0.05, **P < 0.001 (unpaired two-tailed Student’s *t*-test). FITC: Fluorescein isothiocyanate; IL-1β: interleukin-1β; IκBα: inhibitor of nuclear factor kappa B; LPS: lipopolysaccharide; NF-κB: nuclear factor-kappa B; p-IκBα: phospho-inhibitor of NF-κB; PI: propidium iodide; qRT-PCR: quantitative reverse transcription polymerase chain reaction; TNF-α: tumor necrosis factor-α.

Microglia are believed to regulate neuronal responses and fate by communicating with neurons, which affects AD progression. We constructed an *in vitro* neuroinflammation model by stimulating RAW264.7 or BV2 cells with LPS for 24 hours; the LPS treatment group showed increased cell viability and upregulated inflammatory factors in RAW264.7 and BV2 cells (compared with the non-LPS treatment group, *P* < 0.001; **Additional Figure 1A–F**). The cells were then co-cultured with HT22 cells for 24 hours (**Figure 1A**). In the two co-culture models, HT22 cell viability was significantly decreased compared with that in the non-LPS treatment group (*P* < 0.001; **Figure 1B**). In the *in vivo* environment of AD, microglial activation leads to inflammatory factor release, resulting in neuronal damage and apoptosis (Qing et al., 2017). Consistent with this phenomenon, interleukin (IL)-1β, tumor necrosis factor (TNF)-α, and IL-6 expressions were significantly increased in the two neuroinflammation co-culture models compared with those in the non-LPS treatment group. In the HT22–RAW264.7 model, IL-1β, TNF-α, and IL-6 expressions were significantly increased compared with those in the non-LPS treatment group (*P* < 0.001; **Figure 1C**). Similarly, in the HT22–BV2 model, IL-1β, TNF-α, and IL-6 expressions were significantly increased compared with those in the non-LPS treatment group (*P* < 0.001; **Figure 1C**).

To investigate the numbers of apoptotic HT22 cells in the co-culture system, the fluorescence intensities of Annexin V-FITC^+^ and PI^+^ cells were measured. In the HT22–RAW264.7 model, the fluorescence intensities of Annexin V-FITC^+^ cells and PI^+^ cells were significantly increased compared with those in the non-LPS treatment group (*P* < 0.001; **Figure 1D**). In the HT22–BV2 model, the fluorescence intensities of Annexin V-FITC^+^ cells and PI^+^ staining were significantly increased compared with those in the non-LPS treatment group (*P* < 0.001; **Figure 1D**). Moreover, NF-κB, phospho-inhibitor of NF-κB (p-IκBα), and cleaved caspase-3 expressions were significantly elevated (compared with those in the non-LPS treatment group), whereas IκBα expression was attenuated in HT22 cells from both of the co-culture models. In the HT22–RAW264.7 model, NF-κB, p-IκBα, and cleaved caspase-3 expressions were significantly increased after LPS treatment compared with those in the non-LPS treatment group, whereas IκBα expression was significantly decreased (*P* < 0.001; **Figure 1E** and **F**). In the HT22–BV2 model, NF-κB, p-IκBα, and cleaved caspase-3 expressions were significantly increased after LPS treatment compared with those in the non-LPS treatment group, whereas IκBα expression was significantly decreased (*P* < 0.001; **Figure 1G** and **H**). In conclusion, our two *in vitro* neuroinflammation co-culture models effectively simulated the *in vivo* inflammatory environment and exhibited HT22 cell apoptosis, thus providing a robust system for the subsequent experiments.

### miR-146a represses nuclear factor-κB expression and signaling via NR4A3

**Figure 2:**
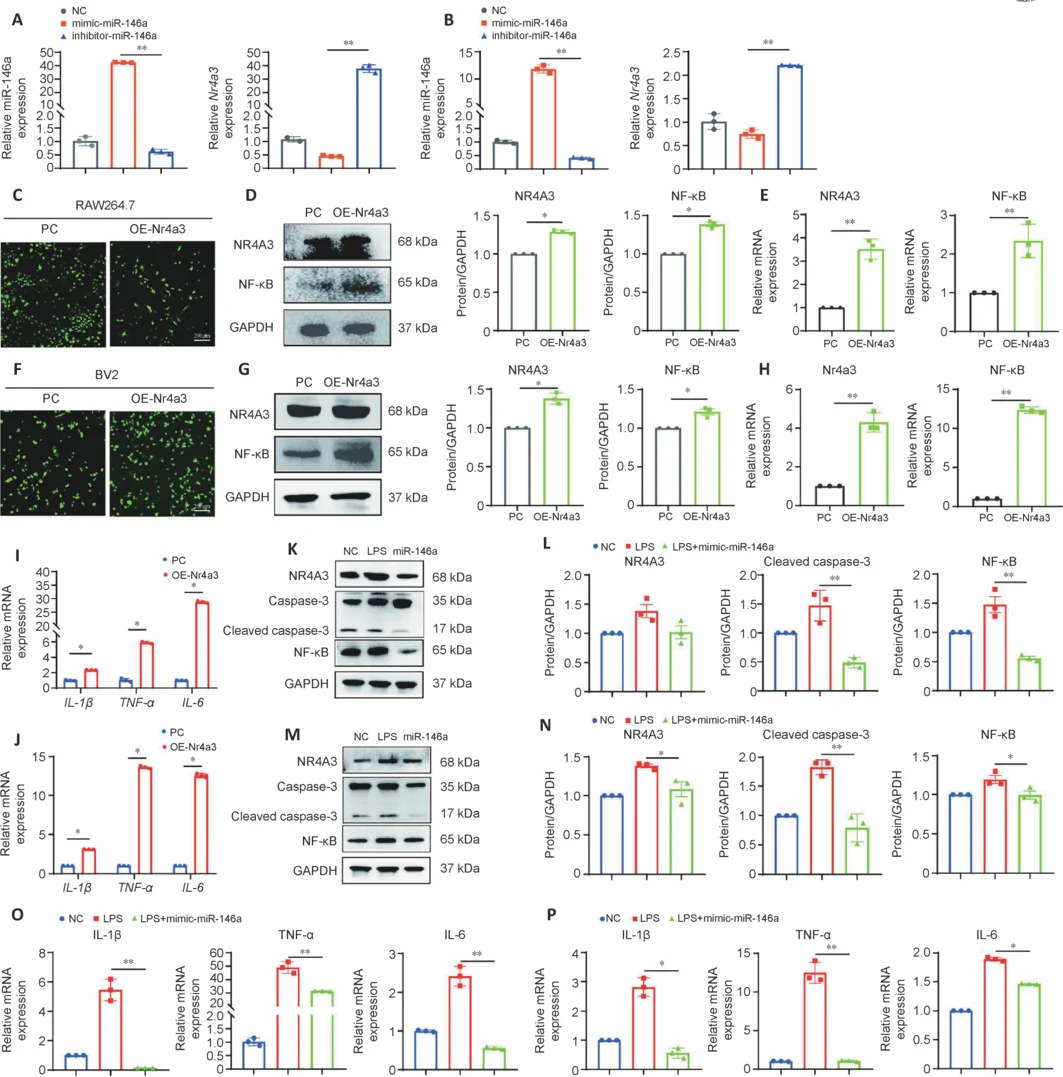
miR-146a inhibits nuclear factor (NF)-κB expression and signaling via nuclear receptor subfamily 4 group A member 3 (NR4A3). (A, B) Quantitative reverse transcription-polymerase chain reaction analysis of miR-146a and *Nr4a3* expression in RAW264.7 (A) and BV2 (B) cells following transient transfection with miR-146a mimic or inhibitor. RAW264.7 and BV2 cells were infected with AAV-NR4a3 to generate OE-Nr4a3 cell lines. (C) Infection efficiency in RAW264.7 cells assessed by fluorescence microscopy. Scale bar: 200 μm. Protein levels of NR4A3 and NF-κB (D) and mRNA expression of NR4A3 and NF-κB (E). (F–H) Infection efficiency in BV2 cells evaluated via GFP fluorescence. Scale bar: 200 μm. Protein expression of NR4A3 and NF-κB (G) and mRNA expression of NR4a3 (H). (I, J) mRNA expression of *IL-1*β, *TNF-α*, and *IL-6* in OE-NR4A3 RAW264.7 (I) and BV2 (J) cells. After LPS treatment, miR-146a was overexpressed. (K–P) Protein levels of NF-κB, caspase-3, and NR4A3 in RAW264.7 (K, L) and BV2 (M, N) cells; mRNA expression of *IL-1*β, *TNF-α*, and *IL-6* in RAW264.7 (O) and BV2 (P) cells. Data are presented as mean ± SEM (*n* = 3). *P < 0.05, **P < 0.01 (one-way analysis of variance with Tukey’s *post hoc* test). AAV: adeno-associated virus; IL-1β: interleukin-1β; IL-6: interleukin-6; NF-κB: nuclear factor-κB; OE: overexpressing; PC: positive control of AAV infected cells without NR4A3 overexpression; TNF-α: tumor necrosis factor-α.

In our previous study, we demonstrated that miR-146a can target *NR4a3* to reduce IL-1β, TNF-α, and IL-6 expressions in retinitis pigmentosa mice (Zhang et al., 2022). To explore whether miR-146a–NR4A3 elicits anti-inflammatory effects in the central nervous system and to investigate the mechanisms by which NR4A3 regulates NF-κB. We transfected RAW264.7 or BV2 cells with miR-146a mimic and miR-146a inhibitor. The qRT-PCR results revealed that miR-146a overexpression resulted in decreased *NR4a3* expression in RAW264.7 and BV2 cells compared with that in the NC group (*P* < 0.001; **Figure 2A** and **B**). By contrast, transfection with the miR-146a inhibitor led to increased *NR4a3* expression in RAW264.7 and BV2 cells compared with that in the NC group (*P* < 0.001; **Figure 2A** and **B**). These results indicate that miR-146a negatively regulates *NR4a3* expression.

The role of NR4A3 in inflammation is controversial. On one hand, NR4A3 reportedly exerts proinflammatory effects in chondrocytes (Ma et al., 2020), but on the other hand, it has been reported to exhibit anti-inflammatory properties in abdominal aortic aneurysm (Qing et al., 2017). To explore the role of NR4A3 in the central nervous system, we used an adeno-associated virus (AAV) method to overexpress NR4A3 in RAW264.7 and BV2 cells using an AAV-OE-*NR4a3* plasmid. Intracellular fluorescence intensity was increased after AAV infection, thus confirming the successful transfection of AAV-OE-*NR4a3* into the cells (**Figure 2C** and **F**). Western blot analysis and qRT-PCR revealed that NF-κB protein and mRNA expressions were significantly increased compared with those in the positive control of AAV infected cells without NR4A3 overexpression (PC) group (protein expression: *P* < 0.001, **Figure 2D** and **G**; mRNA expression: *P* < 0.001, **Figure 2E** and **H**) in RAW264.7 and BV2 OE-NR4A3 cells. The OE-NR4A3 cells also exhibited significantly elevated proinflammatory factor expression. In RAW264.7 OE-NR4A3 cells, IL-1β, TNF-α, and IL-6 expressions were significantly increased compared with those in the PC group (*P* < 0.001; **Figure 2I**). Similarly, in BV2 OE-NR4A3 cells, IL-1β, TNF-α, and IL-6 expressions were significantly increased compared with those in the PC group (*P* < 0.001; **Figure 2J**). These results are consistent with those of Ma et al. (2020), indicating that NR4A3 exerts proinflammatory effects in the central nervous system and upregulates NF-κB to further boost inflammatory factor expression.

To evaluate whether miR-146a can inhibit the secretion of proinflammatory factors by microglia and alleviate the inflammatory environment in AD, we transfected cells with miR-146a mimic according to our previously constructed inflammation model (**Additional Figure 1**). miR-146a overexpression significantly decreased NR4A3, cleaved caspase-3, and NF-κB protein expressions. NR4A3, cleaved caspase-3, and NF-κB protein expressions were significantly decreased in RAW264.7 cells after miR-146a overexpression compared with those in the LPS-treated group (*P* < 0.001; **Figure 2K–N**). Similarly, NR4A3, cleaved caspase-3, and NF-κB protein expressions were significantly decreased in BV2 cells after miR-146a overexpression (*n* = 3) compared with those in the LPS-treated group (*P* < 0.001; **Figure 2K–N**). In addition, IL-1β, TNF-α, and IL-6 expressions were significantly reduced in the miR-146a mimic group compared with those in the LPS group. In RAW264.7 cells, IL-1β, TNF-α, and IL-6 expressions were significantly decreased after miR-146a overexpression compared with those in the LPS-treated group (*P* < 0.001; **Figure 2O**). In BV2 cells, IL-1β, TNF-α, and IL-6 expressions were significantly decreased compared with those in the LPS-treated group (*P* < 0.001; **Figure 2P**). These results indicate that miR-146a may reverse the effects of NR4A3 on NF-κB and proinflammatory factor expression. Together, our findings suggest that miR-146a inhibits NF-κB expression and mitigates the release of proinflammatory cytokines through NR4A3 to prevent cell apoptosis in a neuroinflammatory environment.

### Mesenchymal stem cell–derived extracellular vesicles overexpressing miR-146a are successfully identified and collected

**Figure 3:**
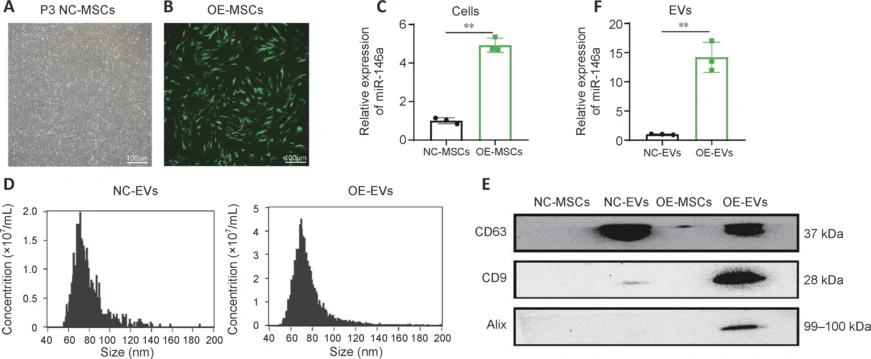
Characterization of MSC-EVs overexpressing miR-146a. (A) Morphology of primary P3 MSCs. Scale bar: 100 μm. (B) Fluorescence microscopy confirming the efficiency of AAV-miR-146a infection in MSCs. Scale bars: 100 μm. (C) qRT-PCR detection of miR-146a expression in NC-MSCs and OE-MSCs. (D) Particle size distribution of EVs (30–150 nm). (E) Western blot showing EV markers (CD63, CD9, and Alix) in MSCs and EVs. (F) qRT-PCR analysis of miR-146a expression in NC-EVs and OE-EVs. Data are presented as mean ± SEM (*n* = 3). **P < 0.001 (unpaired two-tailed Student’s *t*-test). MSC-EVs: mesenchymal stem cell-derived extracellular vesicles; NC: negative control; OE: overexpressing; qRT-PCR: quantitative reverse transcription polymerase chain reaction.

To investigate the anti-inflammatory effects of miR-146a in MSC-EVs, we successfully collected the engineered OE-EVs. We successfully obtained MSCs from human umbilical cords (**Figure 3A**). We did not show the identification of MSCs here because the MSCs were collected as in our previous study (Zhao et al., 2015). Furthermore, we obtained OE-MSCs by infecting cells with AAV-miR-146a (**Figure 3B**). The qRT-PCR results revealed that miR-146a expression was significantly increased to 4.92 ± 0.37-fold in OE-MSCs compared with that in NC-MSCs (*P* < 0.001; **Figure 3C**). Ultrahigh-speed centrifugation was used to separate OE-EVs and NC-EVs, whose particle size distribution was between 40–150 nm (**Figure 3D**). We confirmed the expression of the EV marker proteins CD63, CD9, and ALIX using western blot analysis (**Figure 3E**). To confirm that OE-EVs secreted by OE-MSCs were rich in miR-146a, we performed qRT-PCR; miR-146a expression was significantly increased in OE-EVs compared with that in NC-EVs (*P* < 0.001; **Figure 3F**). In summary, we successfully obtained OE-EVs enriched with miR-146a for our subsequent experiments.

### Mesenchymal stem cell–derived extracellular vesicles overexpressing miR-146a protect hippocampal neurons against inflammation *in vitro*

**Figure 4:**
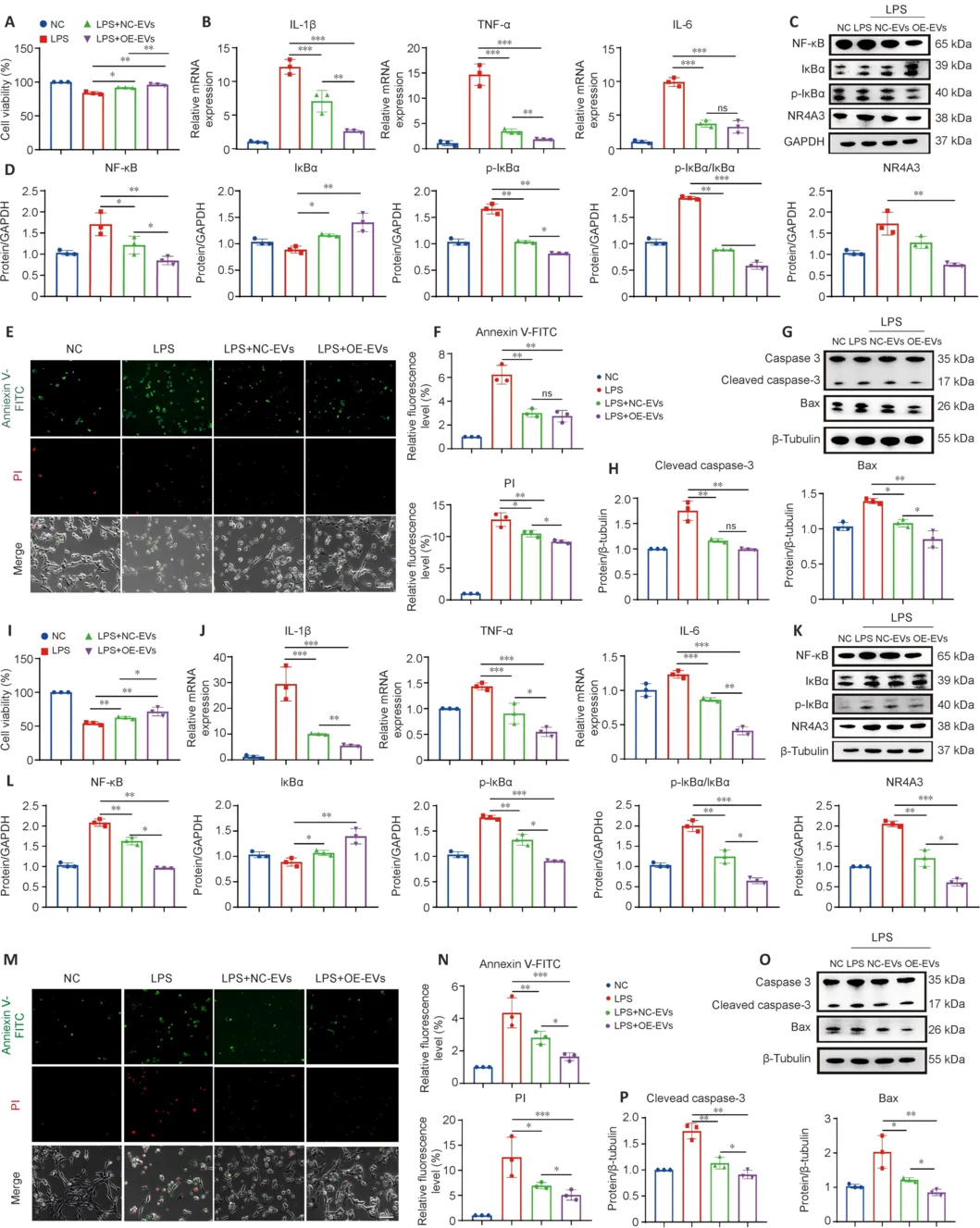
MSC-EVs overexpressing miR-146a protect hippocampal neurons against inflammation *in vitro*. (A, I) Viability of HT22 cells in HT22–RAW264.7 (A) and HT22–BV2 (I) co-culture systems, assessed by CCK-8 assay. (B, J) mRNA expression of IL-1β, TNF-α, and IL-6 in HT22 cells co-cultured with RAW264.7 (B) or BV2 (J) cells. (C, D, K, L) Protein expression and quantification of NF-κB, IκBα, p-IκBα, NR4A3, and GAPDH in HT22–RAW264.7 (C, D) and HT22–BV2 (K, L) systems. (E, F, M, N) Annexin V-FITC/PI staining and fluorescence quantification in HT22–RAW264.7 (E, F) and HT22–BV2 (M, N) systems. (G, H, O, P) Protein expression and quantification of caspase-3, cleaved caspase-3, Bax, and β-tubulin. Data are presented as mean ± SEM (*n* = 3). *P < 0.05, **P < 0.01 (one-way analysis of variance with Tukey’s *post hoc* test). CCK-8: Cell Counting Kit-8; GAPDH: glyceraldehyde-3-phosphate dehydrogenase; IL-1β: interleukin-1β; IL-6: interleukin-6; MSC-EVs: mesenchymal stem cell–derived extracellular vesicles; NF-κB: nuclear factor-kappa B; NR4A3: nuclear receptor subfamily 4 group A member 3; p-IκBα: phospho-inhibitor of NF-κB.

To explore the protective effects of OE-EVs, they were applied in the co-culture systems. The OE-EVs exhibited a very strong protective effect on HT22 cell survival in our two neuroinflammation co-culture models. In HT22-RAW264.7 cells, the HT22 cell survival rate was 91.22% in the LPS-treated group, and significantly increased to 94.32% after OE-EV treatment (*P* < 0.001; **Figure 4A**). In HT22-BV2 cells, the HT22 cell survival rate was 60.37% in the LPS-treated group, and significantly increased to 71.4% after OE-EV treatment (*P* < 0.001; **Figure 4A**). The qRT-PCR results demonstrated that treatment with both EVs significantly reduced the mRNA expressions of proinflammatory factors in HT22 cells in the neuroinflammation models, with OE-EVs eliciting more potent anti-inflammatory effects than NC-EVs. In HT22–RAW264.7 cells, IL-1β, TNF-α, and IL-6 expressions were significantly decreased after OE-EV treatment compared with those in the LPS-treated group (*P* < 0.001; **Figure 4B**). Moreover, OE-EV treatment significantly downregulated NF-κB, p-IκBα, and NR4A3 expressions while increasing-IκBα expression. NF-κB, p-IκBα, and NR4A3 expressions was significantly decreased after OE-EV treatment compared with the LPS-treated group (*P* < 0.001; **Figure 4C a**nd **D**), whereas IκBα expression was significantly increased (*P* < 0.001; **Figure 4C** and **D**). Similar results were also observed in the HT22–BV2 system; IL-1β, TNF-α, and IL-6 expressions were significantly decreased after OE-EV treatment compared with those in the LPS-treated group (*P* < 0.001; **Figure 4I–L**). Moreover, NF-κB, p-IκBα, and NR4A3 expressions were significantly decreased after OE-EV treatment compared with those in the LPS-treated group (*P* < 0.001; **Figure 4B**), whereas IκBα expression was significantly increased (*P* < 0.001; **Figure 4C** and **D**). Thus, OE-EVs greatly suppressed the inflammatory response.

Furthermore, NC-EVs and OE-EVs protected hippocampal neurons against cellular death, which was reflected by Annexin V-FITC/PI staining and western blot analysis of apoptotic proteins. In the HT22–RAW264.7 system, Annexin V-FITC^+^ fluorescence intensity was significantly decreased in the OE-EV-treated group compared with that in the LPS-treated group (*P* < 0.001; **Figure 4E** and **F**). Similarly, OE-EVs reduced PI^+^ fluorescence compared with that in the NC group (*P* < 0.001; **Figure 4E** and **F**). In HT22–BV2 cells, OE-EVs significantly reduced Annexin V-FITC^+^ fluorescence intensity compared with that in the LPS-treated group (*P* < 0.001; **Figure 4E** and **F**). Similarly, OE-EVs reduced PI+ fluorescence intensity compared with that in the LPS-treated group (*P* < 0.001; **Figure 4M** and **N**). Western blot analysis revealed that treatment with both EVs led to downregulation of the apoptosis-related proteins caspase-3 and apoptosis regulator BAX; OE-EVs produced the more significant effect. In the HT22–RAW264.7 system, cleaved caspase-3 and BAX expressions were significantly decreased after OE-EV treatment compared with those in the LPS-treated NC group (*P* < 0.001; **Figure 4G** and **H**). In the HT22–BV2 system, cleaved caspase-3 and BAX expressions were significantly decreased after OE-EV treatment compared with those in the LPS-treated group (*P* < 0.05; **Figure 4O** and **P**). Collectively, these findings suggest that OE-EVs can significantly alleviate the inflammatory environment in the neuroinflammatory co-culture system, thereby protecting neurons and inhibiting apoptosis.

### Mesenchymal stem cell–derived extracellular vesicles overexpressing miR-146a improve cognitive function in 5×FAD mice

After observing the satisfactory efficacy and anti-inflammatory efficiency of OE-EVs in our *in vitro* co-culture model, we investigated whether similar therapeutic effects can be achieved in AD mice. We chose the 5×FAD mouse model, which shows many distinct pathological features of progressive AD at 4 months of age, such as neuroinflammation, Aβ deposition, synaptic plasticity impairment, and cognitive dysfunction (Kosel et al., 2020).

Importantly, crossing the blood–brain barrier is an important challenge for drug delivery in brain diseases. The bioavailability of current intravenous and oral drug therapies to the brain is low. Long-term invasive methods, such as tail vein administration, gavage, brain stereotaxic delivery, and intraperitoneal injection, are therefore not advisable for chronic diseases of the central nervous system such as AD. Thus, intranasal delivery has emerged as a promising non-invasive alternative that bypasses the blood–brain barrier and directly reaches the brain through the olfactory bulb and trigeminal pathways (Kashyap and Shukla, 2019). A recent study has reported the clinical safety and efficacy of intranasal MSC-EV delivery in AD patients (Xie et al., 2023). We therefore administered PKH26-labeled EVs to wild-type mice via nasal drops. Immunofluorescence results revealed a strong fluorescence signal of EV uptake in the cornu ammonis 1 (CA1) region of the hippocampus (**Additional Figure 2D** and **E**), and the EVs were taken up by ionized calcium-binding adaptor molecule 1 (Iba1)-labeled microglia in the CA1 region (**Additional Figure 2F**). These findings indicate that MSC-EVs can be delivered into the mouse brain, and especially the hippocampal region, through nasal drops.

**Figure 5:**
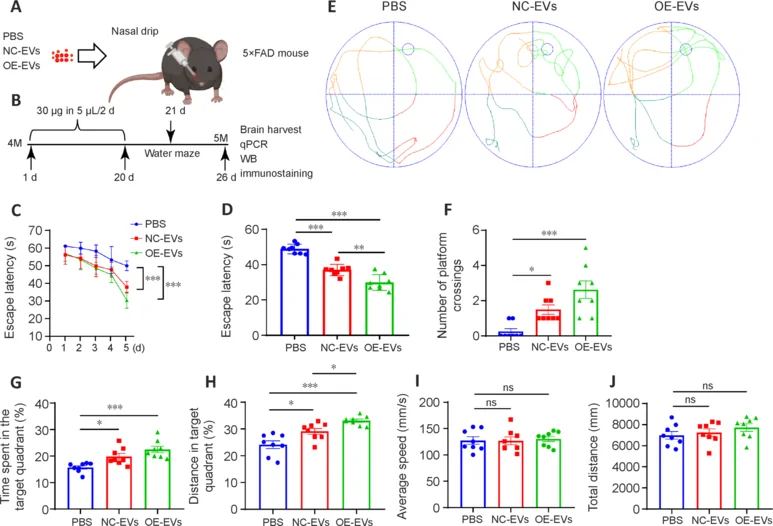
Mesenchymal stem cell-derived EVs overexpressing miR-146a improve cognitive function in 5×FAD mice. The mice received intranasal administration of PBS, NC-EVs, or OE-EVs. (A, B) Schematic of the therapeutic regimen in 5×FAD mice. (C–J) Escape latency during 5-day training (C), escape latency on test day (D), representative swimming paths (E), number of platform crossings (F), time spent in the target quadrant (G), distance traveled in the target quadrant (H), average swimming speed (I), and total distance traveled (J) tested using the Morris water maze. Data are presented as mean ± SEM (*n* = 8). *P < 0.05, **P < 0.001, ***P < 0.0001 (one-way analysis of variance with Tukey’s test [D, F–J]; two-way analysis of variance with Bonferroni correction [C]). EVs: Extracellular vesicles; NC: negative control; NC-EVs: normal MSC-EVs; ns: not significant; OE: overexpressing; OE-EVs: MSC-EVs overexpressing miR-146a; PBS: phosphate-buffered saline; qPCR: quantitative polymerase chain reaction; WB: western blotting.

In the current study, mice were intranasally administered NC-EVs or OE-EVs (30 μg) in 5 μL of PBS every 2 days (**Figure 5A** and **B**). After 10 days of treatment, all three groups of mice were subjected to a 1-week Morris water maze test. During the 5-day visible platform training phase, all three groups of mice were able to find the platform. However, the time taken to reach the platform was significantly shorter in mice in the OE-EV treatment group than in mice in the PBS-treated group (*P* < 0.001; **Figure 5C–E**). After the platform was removed, the number of times crossing the platform position, the time spent in the platform quadrant, and the distance traveled in the platform quadrant were all significantly higher in the OE-EV treatment group than in the PBS-treated group (*P* < 0.001; **Figure 5F–H**). In addition, there were no significant differences in total distance, swimming distance, and speed among the groups in all trials, indicating unimpaired swimming ability in all groups (*P* < 0.001; **Figure 5I** and **J**). The 5×FAD mice treated with OE-EVs tended to spend more time in the target quadrant, in which the hidden platform was located, indicating improved learning and memory (**Additional Figure 3B**). Treatment with OE-EVs also alleviated the escape latency during the Morris water maze test in 5×FAD mice (**Additional Figure 3A–F**). These findings strongly suggest that OE-EV treatment markedly improves cognitive deficits in AD mice.

### Mesenchymal stem cell–derived extracellular vesicles overexpressing miR-146a suppress glial cell activation and alleviate Alzheimer’s disease progression

**Figure 6:**
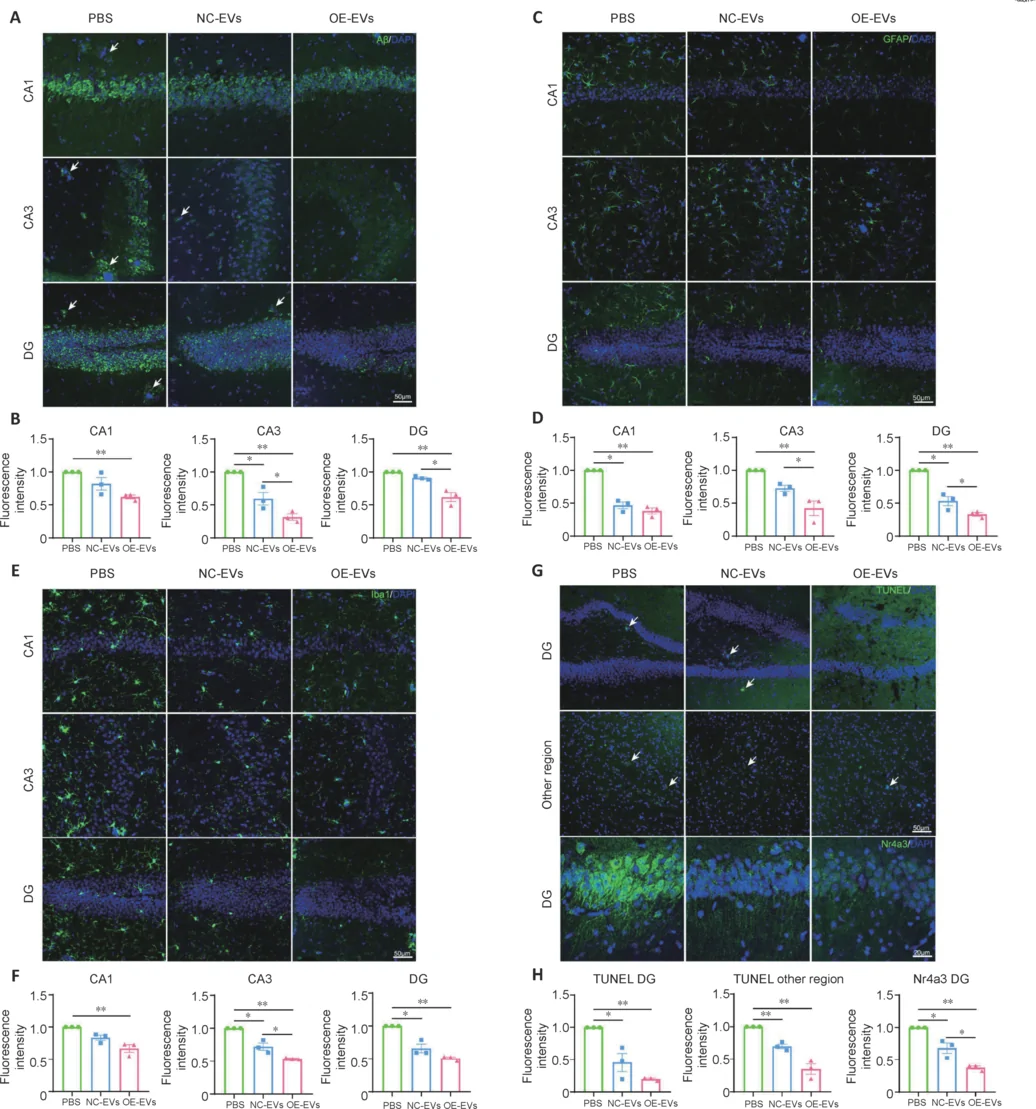
MSC-EVs overexpressing miR-146a suppress glial activation and ameliorate AD pathology. (A) Brain sections stained with DAPI (blue) and Aβ (green). Aβ deposition was elevated in the hippocampal CA1, CA3, and DG regions but was reduced by OE-EVs. Scale bar: 50 μm. White arrows indicate Aβ deposits. (B) Quantification of Aβ fluorescence intensity. (C) GFAP staining (green) showing astrocyte activation; OE-EVs reduced GFAP expression. Scale bar: 50 μm. (D) Quantification of GFAP intensity. (E) Iba1 staining (green) indicating microglial activation; OE-EVs suppressed activation. Scale bar: 50 μm. (F) Quantification of Iba1 intensity. (G) TUNEL (green) and NR4A3 (green) staining. Apoptotic cells and NR4A3 expression were increased in AD mice and reduced by OE-EVs. Scale bars: TUNEL, 50 μm; NR4A3, 20 μm. White arrows indicate TUNEL^+^ cells. (H) Quantification of TUNEL and NR4A3 fluorescence. Data are presented as mean ± SEM (*n* = 3). *P < 0.05, **P < 0.001 (one-way analysis of variance with Tukey’s *post hoc* test). AD: Alzheimer’s disease; Aβ: amyloid-beta; DAPI: 4′,6-diamidino-2-phenylindole; DG: dentate gyrus; GFAP: glial fibrillary acidic protein; EVs: extracellular vesicles; Iba1: ionized calcium-binding adapter molecule 1; MSC-EVs: mesenchymal stem cell-derived EVs; NC: negative control; OE: overexpressing; TUNEL: terminal deoxynucleotidyl transferase dUTP nick end labeling.

To elucidate the protective effects of OE-EVs in the AD mouse model, mouse brain sections were subjected to immunofluorescence staining to evaluate the distribution of Aβ deposits, hippocampal neuronal apoptosis, and glial cell activation in the hippocampi of AD mice. Treatment with EVs decreased Aβ aggregates in the CA1, CA3, and dentate gyrus (DG) regions of the hippocampus; OE-EVs produced a more significant decline. The relative fluorescence intensity of Aβ aggregates in the CA1, CA3, and DG regions was significantly decreased after OE-EV treatment compared with that in the PBS-treated group (*P* < 0.001; **Figure 6A** and **B**). After EV treatment, the relative fluorescence intensity of glial fibrillary acidic protein (GFAP), an astrocyte activation marker, was significantly decreased in the CA1, CA3, and DG regions. The relative fluorescence intensity of GFAP in the CA1, CA3, and DG regions was significantly decreased after OE-EV treatment compared with that in the PBS-treated group (*P* < 0.001; **Figure 6C** and **D**). Similarly, the microglial marker Iba1 was significantly downregulated in the CA1, CA3, and DG regions after EV treatment. The relative fluorescence intensity of Iba1 in the CA1, CA3, and DG regions was significantly decreased after OE-EV treatment compared with that in the PBS-treated group (*P* < 0.01; **Figure 6E** and **F**). In addition, the fluorescence intensity of the TUNEL^+^ staining area was significantly decreased in the different hippocampal regions after OE-EV treatment. In the DG region, the relative fluorescence intensity of TUNEL+ staining was significantly decreased after OE-EV treatment compared with that in the PBS-treated group (*P* < 0.01; **Figure 6G** and **H**). In other regions, the relative fluorescence intensity of TUNEL^+^ staining was significantly decreased after OE-EV treatment compared with the PBS-treated group (*P* < 0.01; **Figure 6G** and **H**). Surprisingly, NR4A3 expression was significantly decreased after OE-EV treatment compared with that in the PBS-treated group (*P* < 0.01; **Figure 6G** and **H**). These results suggest the protective effect of OE-EVs on neuronal apoptosis, and indicate that OE-EVs may suppress glial cell activation and alleviate AD progression *in vivo*.

### In 5×FAD mice, mesenchymal stem cell–derived extracellular vesicles overexpressing miR-146a protect hippocampal neurons against inflammation though NR4A3

**Figure 7:**
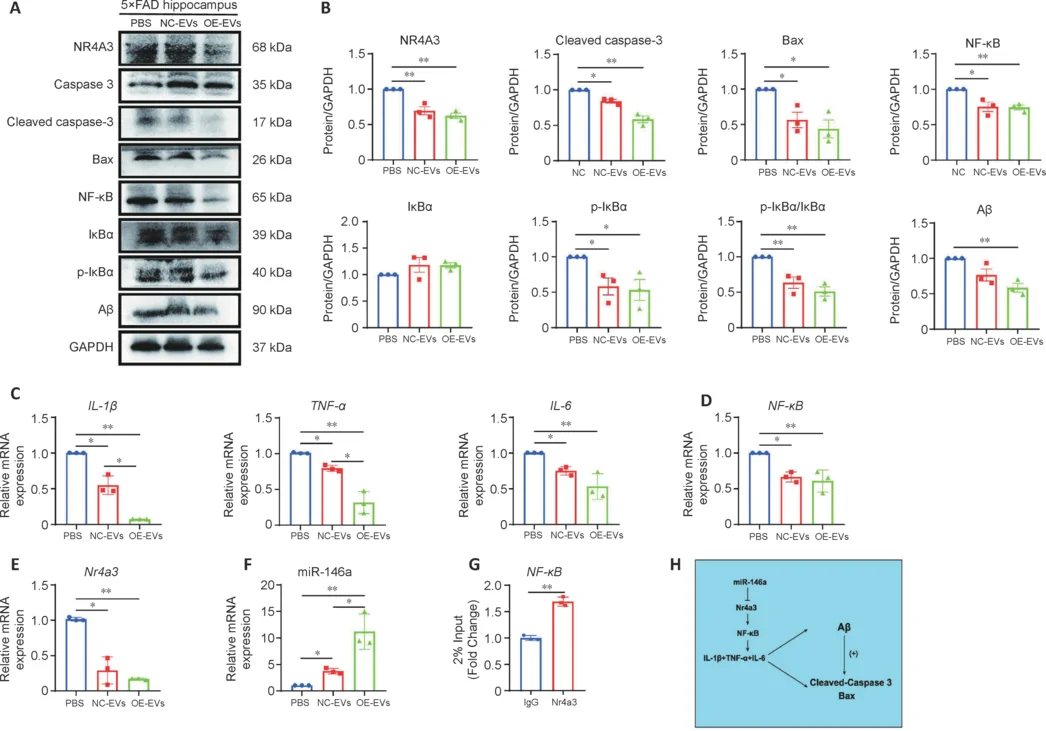
MSC-EVs overexpressing miR-146a attenuate neuroinflammation in 5×FAD mice. (A, B) Protein levels and quantification of NR4A3, caspase-3, cleaved caspase-3, Bax, NF-κB, IκBα, p-IκBα, and Aβ. (C–G) mRNA expression of *IL-1*β, *TNF-α*, *IL-6* (C), *NF-κB* (D), *Nr4a3* (E), miR-146a (F), and *NF-κB* (G). (H) Schematic of the molecular mechanisms regulated by OE-EVs in macrophages, microglia, and neurons. Data are presented as mean ± SEM (*n* = 3). *P < 0.05, **P < 0.01 (one-way analysis of variance with Tukey’s *post hoc* test). EVs: Extracellular vesicles; IL-1β: interleukin-1β; IL-6: interleukin-6; MSC-EVs: mesenchymal stem cell-derived EVs; NC: negative control; NF-κB: nuclear factor-kappa B; NR4A3: nuclear receptor subfamily 4 group A member 3; OE: overexpressing; TNF-α: tumor necrosis factor-α.

To investigate the protective mechanisms of the signaling pathway of OE-EVs *in vivo*, western blot analysis and qRT-PCR were used to explore the apoptosis and NF-κB signaling pathways as well as the expression of proinflammatory factors, Aβ, phosphorylated (p)-tau, and NR4A3. Western blot analysis revealed that cleaved caspase-3 and BAX expressions in the hippocampi of AD mice were significantly attenuated after EV treatment. Cleaved caspase-3, BAX, NF-κB, p-IκBα, and NR4A3 expressions were significantly decreased after OE-EV treatment compared with those in the PBS-treated group (*P* < 0.05; **Figure 7A** and **B**). Similarly, expression of the pathological protein Aβ was significantly decreased after OE-EV treatment compared with that in the PBS-treated group (*P* < 0.05; **Figure 7A** and **B**). Although the expression of p-tau was also significantly attenuated after EV treatment, this effect was not observed in the hippocampal region (**Additional Figure 4**). qRT-PCR revealed that proinflammatory factor expression was significantly decreased in the hippocampus, whereas miR-146a expression was significantly elevated. IL-1β, TNF-α, and IL-6 expressions were significantly decreased after OE-EV treatment compared with those in the PBS-treated group (*P* < 0.05; **Figure 7C**). Moreover, NF-κB and NR4A3 expressions were significantly decreased after OE-EV treatment compared with those in the PBS-treated group (*P* < 0.05; **Figure 7D** and **E**). Additionally, miR-146a expression was significantly increased after OE-EV treatment compared with the PBS-treated group (*P* < 0.05; **Figure 7D–F**). These *in vivo* experimental results were consistent with the *in vitro* results.

Furthermore, ChIP-PCR was used to investigate NR4a3-regulated inflammation through the activation of NF-κB transcription. Using bioinformatic analysis, we predicted that NR4a3 can bind to one site of the NF-κB promoter region: AAGGTCA. We again used NR4a3-overexpressing BV2 cells to observe whether NR4A3 can bind to the NF-κB promoter. NR4A3 significantly promoted NF-κB transcription compared with immunoglobulin G (*P* < 0.05; **Figure 7G**). In conclusion, OE-EV treatment significantly downregulated NR4A3 in glial cells of the AD mouse brain and suppressed NF-κB activation, thereby mitigating the release of inflammatory factors and the deposition of pathological proteins in neurons (**Figure 7H**).

## Discussion

### Neuroprotective effects of mesenchymal stem cell–derived extracellular vesicles overexpressing miR-146a in the hippocampus in Alzheimer’s disease

To date, conventional therapies and common drug therapies have very limited efficacy to clinically treat AD. Stem cell-derived exosome therapy is a new, safe, and effective treatment modality in the clinic (Xia et al., 2022). In various cell models of AD, MSC-EVs/exosomes effectively protect neurons against Aβ toxicity, inhibit neuronal apoptosis, and promote neuronal migration and the growth of neuronal processes (Lee et al., 2018; Wei et al., 2020; Chen et al., 2021). In the present study, we constructed a co-culture system of macrophages/microglia and HT22 neurons. LPS was used to activate the macrophages/microglia, which resulted in apoptosis and a decrease in cell viability of HT22 neurons. The administration of NC-EVs and OE-EVs significantly increased HT22 neuron viability, significantly decreased the fluorescence intensity of Annexin V-FITC^+^/PI^+^ cells, and significantly downregulated the apoptosis-related proteins cleaved caspase-3 and BAX. These findings are consistent with previous reports on the effects of MSC-EVs/exosomes on neural cells (Lee et al., 2018; Wei et al., 2020; Chen et al., 2021). Stem cell–derived EVs/exosomes reportedly improve cognitive function and delay AD progression in transgenic mice (Chen et al., 2021), APPSweInd mice (Yuyama et al., 2014), APP/PS1 mice (Wang et al., 2018; Nakano et al., 2020a), 5×FAD mice (Apodaca et al., 2021; Gao et al., 2023), and streptozotocin-induced AD-like mice (Liu et al., 2022). Most studies have used intracranial or intravenous delivery methods (Chavan et al., 2023), including intracerebroventricular injection, substantia nigra pars compacta and striatum injection, bilateral injection into the lateral ventricles, brain infusion, single retro-orbital vein injection, and caudal vein injection. In the current study, NC-EVs and OE-EVs effectively enhanced the learning and memory abilities of 5×FAD mice, reduced the numbers of TUNEL+ cells in the hippocampus, attenuated Aβ and p-tau expression in the brain, inhibited the proliferation and activation of astrocytes and microglia, and downregulated cleaved caspase-3 and BAX in the hippocampi of 5×FAD mice. Unlike in previous studies, we used intranasal delivery, and demonstrated that MSC-EVs reached almost all brain regions—including the hippocampus—using this mode of delivery. In summary, our results suggest that NC-EVs and OE-EVs can improve cognitive function and reduce neuronal apoptosis in the brains of 5×FAD mice.

### Mesenchymal stem cell–derived extracellular vesicles overexpressing miR-146a alleviate Alzheimer’s disease lesions by inhibiting neuroinflammation

In recent years, clinical studies into AD treatments have focused on reducing Aβ plaques using anti-Aβ antibodies, inhibitors of β-site APP cleaving enzyme, and γ-secretase inhibitors (Tayeb et al., 2012; Madrasi et al., 2021). Unfortunately, these strategies have failed to reduce plaque in clinical trials. Targeting plaque reduction may therefore not be a good approach for ameliorating cognitive decline. In addition, the pathogenesis of AD is complex, involving neuroinflammation, metabolic disorders, gene mutations, neurotoxic damage, and oxidative stress (Fang et al., 2019). Accumulating evidence indicates that any strategy for the treatment of AD should possess potent anti-inflammatory and neuroprotective properties (Akiyama et al., 2000). Previous studies have demonstrated that stem cell-derived EVs/exosomes do not produce any adverse side effects (Campanella et al., 2019) but exert anti-inflammatory effects to effectively treat various neurodegenerative diseases (Chavan et al., 2023).

Stem cell-derived EVs/exosomes play an anti-inflammatory role in retinal degenerative diseases by targeting the microglia and monocytes/macrophages (Yu et al., 2016). They significantly inhibit the inflammasome, NF-κB signaling activation, and the expression of proinflammatory factors such as monocyte chemoattractant protein-1a, IL-6, TNF-α, IL-1β, and cyclooxygenase-2 (Zhang et al., 2019, 2022; Bian et al., 2020). The anti-inflammatory effects of stem cell-derived EVs/exosomes have also been reported in other neurodegenerative diseases. For example, in an animal model of Parkinson’s disease, stem cell-derived EVs/exosomes attenuate neurotoxicity and neuroinflammation and suppress autophagy and pyroptosis by targeting cyclin-dependent kinase 5 and NLR family pyrin domain-containing 3 (Kojima et al., 2018; Li et al., 2021). Wang et al. (2018) reported that MSC-EVs improve hippocampal function in APP/PS1 mice by inhibiting inducible nitric oxide synthase (iNOS) expression and mitigating neuroinflammation. Apodaca et al. (2021) demonstrated that treating 5×FAD mice with MSC-EVs suppresses microglial activation and downregulates proinflammatory and immune response-related genes as well as the proinflammatory factors interferon-γ and IL-17. Zhai et al. (2021) reported that MSC-exosomes improve neurological function in APP/PS1 mice by inhibiting microglial activation, proinflammatory factor release, and neuronal death. Liu et al. revealed that MSC-exosomes alleviate cognitive decline in streptozotocin-induced AD-like mice by upregulating brain-derived neurotrophic factor to inhibit inflammatory factor release and microglial activation (Liu et al., 2022). Gao et al. (2023) demonstrated that neuroblastoma cell-derived EVs improve cognitive function in 5×FAD mice and effectively allay disease progression by suppressing the genes involved in Aβ protein synthesis; the proinflammatory factors CD86, iNOS, and IL-1β; and microglial activation. Together, these studies highlight the anti-inflammatory effects of EVs/exosomes.

In the present study, OE-EVs significantly inhibited neuroinflammation both *in vivo* and *in vitro*. *In vitro* experiments revealed that OE-EVs not only blunted the inflammatory response in macrophages/microglia but also inhibited expression of the proinflammatory factors IL-1β, IL-6, and TNF-α in hippocampal neurons. Furthermore, they inhibited NF-κB signaling activation and the expressions of cleaved caspase-3, BAX, and their corresponding signaling pathways. The activation of microglia and astrocytes was also suppressed in 5×FAD mice treated with OE-EVs. Moreover, proinflammatory factor expression, NF-κB signaling, and the number of TUNEL^+^ hippocampal neurons were also attenuated, whereas the cognitive function of mice was significantly enhanced. In summary, our results suggest that MSC-EVs alleviate AD progression by inhibiting neuroinflammation.

Dabrowska et al. (2020) reviewed many similar studies, but many of them did not identify the key effective components within stem cell-derived EVs/exosomes that regulate the occurrence and progression of neuroinflammation, nor did they uncover the molecules being targeted. In the current study, we identified miR-146a as a key effective component within MSC-EVs that targets the transcription factor *Nr4a3* to regulate NF-κB signaling, which eventually mitigates neuroinflammation.

**miR-146a regulates nuclear factor-κB signaling activation through NR4A3**

miRNAs are a group of conserved small noncoding RNAs that participate in the occurrence and development of diseases by targeting and inhibiting mRNA transcription (Cone et al., 2021). miRNAs also play an important role in regulating brain development and adult neuronal cell function (Laurent et al., 2018). miR-146a has been widely implicated in inflammatory conditions of the central nervous system (Olivieri et al., 2021). In clinical studies, miR-146a was revealed to be upregulated in the blood of AD patients (Ansari et al., 2019; Lei et al., 2021; Gong et al., 2022) and in various types of inflammation (Banerjee et al., 2013; Bai et al., 2018). Conversely, many studies have confirmed the anti-inflammatory roles of miR-146a in AD (Mai et al., 2019; Nakano et al., 2020a, b; Lei et al., 2021; Liang et al., 2021; Ma et al., 2021; Yang et al., 2021; Zhan-Qiang et al., 2023), despite its upregulation. In the present study, MSC-EVs carrying miRNA-146a downregulated the inflammatory factors IL-1β, IL-6, and TNF-α, suppressed NF-κB signaling, alleviated neuronal apoptosis, and protected neurons in both *in vitro* and *in vivo* models. These findings seem to be contradictory; however, our recent study on retinal degenerative disease revealed that the inflammation-induced upregulation of miR-146a might be a form of feedback regulation, serving as an innate protective mechanism for tissues and organs (Zhang et al., 2022). The mechanisms underlying this feedback regulation of inflammation will be investigated in a future study.

NR4A3 is also known as NOR-1; its natural ligands have not yet been identified (Wang et al., 2003). NR4A3 overexpression activates NF-κB signaling and *vice versa* (Ma et al., 2020). Ma et al. (2020) reported that NR4A3 overexpression in chondrocytes enhances the IL-1β-mediated expression of cartilage matrix-degrading enzymes such as matrix metalloproteinase-3 and -9, iNOS, and cyclooxygenase-2. Using cecal ligation and puncture, Gao et al. established a sepsis model in which miR-501-5p, which was downregulated in the septic myocardium, was found to target NR4a3 and inhibit its binding to Bcl-2, thereby alleviating myocardial cell apoptosis and inflammation (Gao et al., 2022). NR4A3 is also reported to stimulate NF-κB transcription in atherosclerosis using ChIP-PCR (Zhao et al., 2010). In the present study, we also revealed that NR4A3 regulates NF-κB expression. We successfully transduced RAW264.7 and BV2 cells with lentivirus and validated the infection efficiency using western blot analysis and qRT-PCR. NR4A3 overexpression significantly upregulated NF-κB at both the mRNA and protein levels, and this was accompanied by the increased expressions of the inflammatory factors IL-1β, IL-6, and TNF-α. We then confirmed that NR4A3 promoted inflammation through the transcriptional activation of NF-κB. The present study also demonstrated that LPS stimulation in the co-culture system upregulated NR4A3 and NF-κB in hippocampal neurons, thus promoting their apoptosis; however, these effects were reversed by overexpressing miR-146a. In our previous study, we demonstrated for the first time that miR-146a protects retinal neurons by targeting *Nr4a3* to inhibit IL-1β, IL-6, and TNF-α expressions and suppress NF-κB signaling (Zhang et al., 2022). Collectively, our results suggest that miR-146a regulates NF-κB signaling activation through the transcription factor NR4A3 to inhibit hippocampal inflammation and mitigate neuronal apoptosis in AD mice.

## Limitations

Although stem cell–derived EVs may have great potential for the clinical treatment of neurological disorders, some limitations remain. In the current study, we used an intranasal dose of EVs (10 μg/μL) and evaluated their brain distribution at a single time point (24 hours post-administration), which restricted our ability to assess the dose–response effects and biodistribution dynamics over time. In addition, the lack of standardization for the use of clinical grade stem cell-derived EVs makes them challenging to use in clinical treatment.

## Conclusions

The present findings indicate that the intranasal administration of OE-EVs can ameliorate hippocampal neuronal apoptosis, promote cognitive recovery, and delay the pathological process of AD both *in vitro* and *in vivo*. OE-EVs protected hippocampal neurons mainly by regulating the expression of NR4A3 to inhibit NF-κB signaling, which attenuated the release of proinflammatory factors. Hence, OE-EVs may be potential therapeutic drugs for the clinical treatment of neurodegenerative diseases.

**Additional files:**

***Additional Figure 1:*** *LPS upregulates inflammatory factor expression in macrophages/microglia.*

***Additional Figure 2:*** *Uptake of intranasally delivered MSC-EVs in the hippocampus of WT mice.*

***Additional Figure 3:*** *MSC-EVs overexpressing miR-146a reduce escape latency of mice in the Morris water maze.*

***Additional Figure 4:*** *MSC-EVs overexpressing miR-146a suppress p-Tau expression in 5×FAD mice.*

***Additional Table 1:*** *Antibody information.*

**Additional Table 1:** Antibody information

Antibodies	Dilutions	Cat#	RRID	Suppliers	Application
Anti-mouse-IκBα	1:1000	4814	AB_390781	Cell Signaling Technology, Danvers, MA, USA	WB
Anti-rabbit-phospho-IκBα (Ser32)	1:1000	5209	AB_10829358	Cell Signaling Technology	wb
Anti-rabbit-NF-κB p65	1:1000	8242	AB_10859369	Cell Signaling Technology	wb
Anti-rabbit-CD9	1:1000	13403S	AB_2732848	Cell Signaling Technology	wb
Anti-rabbit-CD63	1:1000	55051S	AB_2799476	Cell Signaling Technology	wb
Anti-rabbit-Alix	1:1000	92880S	AB_2800192	Cell Signaling Technology	wb
Anti-rabbit-Caspase 3	1:1000	14220S	AB_2798429	Cell Signaling Technology	wb
Anti-rabbit-NR4A3	1:1000	orb158012	AB_3699163	Biorbyt, Cambridge, UK	IF, wb
Anti-rabbit-GAPDH	1:1000	5174S	AB_10622025	Cell Signaling Technology	wb
Anti-rabbit-β-Tubulin	1:1000	2128S	AB_823664	Cell Signaling Technology	wb
Anti-rabbit-HRP	1:5000	7074	AB_2099233	Cell Signaling Technology	wb
Anti-mouse-HRP	1:5000	7076	AB_330924	Cell Signaling Technology	wb
Anti-rabbit-GFAP	1:2000	ab7260	AB_305808	Abcam, Cambridge, UK	IF
Anti-rabbit-Iba1	1:1000	019-19741	AB_839504	Wako, Osaka, Tokyo	IF
Anti-rabbit-Aβ	1:1000	25524-1-AP	AB_2880118	Proteintech, Wuhan, China	IF, WB
Anti-rabbit-phospho-tau (Thr181) (D9F4G)	1:1000	12885s	AB_2798053	Cell Signaling Technology	IF
Anti-rabbit-Tau(D1M9X)XP	1:1000	46687s	AB_2783844	Cell Signaling Technology	IF
Anti-rabbit-Bax	1:1000	2772	AB_10695870	Cell Signaling Technology	wb
Goat anti-rabbit H&L	1:1000	ab150077	AB_2630356	Abcam	IF
Goat anti-mouse H&L	1:1000	A-11001	AB_2534069	Invitrogen, Carlsbad, CA, USA	IF
Donkey anti-mouse H&L	1:1000	A-21202	AB_141607	Invitrogen	

Aβ: Amyloid-β; GAPDH: glyceraldehyde-3-phosphate dehydrogenase; GFAP: glial fibrillary acidic protein; HRP: horseradish peroxidase; Iba1: ionized calcium-binding adaptor molecule 1; IF: immunofluorescence; IκBα: inhibitor of NF-κB; NF-κB: nuclear factor-kappa B; NR4A3: nuclear receptor subfamily 4 group A member 3; WB: western blotting.

***Additional Table 2:*** *Primers for quantitative reverse transcription-polymerase chain reaction.*

**Additional Table 2:** Primers for quantitative reverse transcription-polymerase chain reaction

Target genes	Forward primer (5'-3')	Reverse primer (5'-3')
IL-1β	TTG TGC TGT GGA GAA GCT GT	AAC GTC ACA CAC CAG CAG GTT
TNF-α	AGC AAA CCA CCA AGT GGA GGA	GCT GGC ACC ACT AGT TGG TTG T
IL-6	TCT ATA CCA CTT CAC AAG TCG GA	GAA TTG CCA TTG CAC AAC TCT TT
NR4a3	ACC GCC ACA CCC TCC CGC GC	CCT TAT AGT CCT TAT CAT CGT C
NF-κB	GAG GTC TTA GTT TGG ACT CTG GT	AGC CAC TTC ACC AGA AGG TTT T
GAPDH	ATG ATT CTA CCC ACG GCA AG	CTG GAA GAT GGT GAT GGG TT
Hsa-U6	GCT TCG GCA GCA CAT ATA CTA AAA T	CTC ACA CCG TGT CGT TCC A
Hsa-miR-146a-5p	AGA ACT GAA TTC CAT GGG TTA A	
mmu-U6	GCT TCG GCA GCA CAT ATA CTA AAA T	
mmu-miR-146a-5p	AGA ACT GAA TTC CAT GGG TTA A	

GAPDH: Glyceraldehyde-3-phosphate dehydrogenase; Has-U6: U6 small nuclear RNA; Hsa-miR-146a-5p: microRNA 146a; IL-1β: interleukin-1β; IL-6: interleukin 6; mmu-miR-146a-5p: microRNA 146a; mmu-U6: U6 small nuclear RNA; NF-κB nuclear factor kappa B; NR4a3: nuclear receptor subfamily 4 group Amember 3; TNF-α: tumor necrosis factor-α.

Additional Figure 1 LPS upregulates inflammatory factor expression in macrophages/microglia.
RAW264.7 and BV2 cells were stimulated with 1 μg/mL LPS for 24 hours. (A) Morphological changes in RAW264.7 cells (red arrows: increased synapses and activation). Scale bar: 200 μm. (B) Cell viability (Cell Counting Kit-8 assay). (C) Proinflammatory cytokine expression (quantitative reverse transcription-polymerase chain reaction). (D) Morphological changes in BV2 cells (red arrows: enlarged cell bodies, shortened protrusions). Scale bar: 200 μm. (E) Viability of BV2 cells. (F) Proinflammatory cytokine expression. Data are presented as mean ± SEM (*n* = 3). ^*^P < 0.05, ^**^P < 0.001 (unpaired two-tailed *t*-test). IL-1β: interleukin-1β; IL-6: interleukin-6; LPS: lipopolysaccharide; NC: negative control; TNF-α: tumor necrosis factor-α.

Additional Figure 2 Uptake of intranasally delivered MSC-EVs in the hippocampus of WT mice.
PKH26-labeled PBS or EVs were administered intranasally to WT mice. (A.F3) Brain sections stained with DAPI (blue) and Iba1 (green). EVs were efficiently taken up in the hippocampal CA1 and other regions, and internalized by Iba1^+^ microglia (F3). DAPI: 4ʹ,6-Diamidino-2-phenylindole; Iba1: ionized calcium-binding adapter molecule 1; MSC-EVs: Mesenchymal stem cell-derived extracellular vesicles; PBS: phosphate-buffered saline; WT: wild-type.

Additional Figure 3 MSC-EVs overexpressing miR-146a reduce escape latency of mice in the Morris water maze.
(A) Division of the maze into quadrants I–IV. (B–E) Escape latency per quadrant over 6 training days. (F) Proportion of time spent in each quadrant. Data are presented as mean ± SEM (*n* = 8). EVs: Extracellular vesicles; MSC-EVs: mesenchymal stem cell-derived EVs; NC: negative control; OE: overexpressing; PBS: phosphate-buffered saline.

Additional Figure 4 MSC-EVs overexpressing miR-146a suppress p-Tau expression in 5×FAD mice.
Brain sections stained with DAPI (blue) and p-Tau (green). p-Tau expression was elevated in the DG and other hippocampal regions but reduced by OE-EVs. DAPI: 4ʹ,6-Diamidino-2-phenylindole; EVs: extracellular vesicles; MSC-EVs: mesenchymal stem cell-derived EVs; NC: negative control; OE: overexpressing.

## Data availability statement:

*All relevant data are within the paper and its Additional files.*
